# A kwashiorkor case due to the use of an exclusive rice milk diet to treat atopic dermatitis

**DOI:** 10.1186/s12937-015-0071-7

**Published:** 2015-08-21

**Authors:** Francesca Mori, Daniele Serranti, Simona Barni, Neri Pucci, Maria Elisabetta Rossi, Maurizio de Martino, Elio Novembre

**Affiliations:** 1 Allergy Unit, Anna Meyer Children’s University Hospital, Viale Pieraccini 24, 50139 Florence, Italy; 2 Department of Health Sciences, University of Florence, Viale Pieraccini 24, 50139 Florence, Italy; 3 Infectious Diseases Unit, Anna Meyer Children’s University Hospital, Viale Pieraccini 24, 50139 Florence, Italy

## Abstract

Although several cases of severe hypoalbuminemia resulting from rice milk have been described in the past, today the use of rice milk without nutritional counseling to treat eczema is still a continuing, poor practice. We describe a kwashiorkor case in an infant with severe eczema exclusively fed with rice milk. It is well documented that rice milk is not a sufficient protein source. Moreover, only a small portion of eczema is triggered by food allergy. In conclusion this case raises the importance of managing dietary changes facing food allergies with responsibility for specialized consensus among pediatricians, nutritionists, endocrinologists and allergists all of them specialist professionals.

## Background

We describe a case of severe hypoalbuminemia provoked by an unnecessary and inappropriate elimination diet based on rice milk in an infant with severe atopic dermatitis (AD), which was thought to be secondary to food allergy.

### Case presentation

An exclusively brestfed boy infant developed AD at the age of 4 months. In the beginning, the eczema was treated with antibiotics, topical steroids and brief courses of oral steroids. The mother was dissatisfied by the outcome of the pediatrician’s advice so she consulted a naturopathic doctor who prescribed a restricted diet.

At 6 months the child’s daily diet consisted of rice milk, fruits, rice poultry and vegetable broth.

**Fig. 1:**
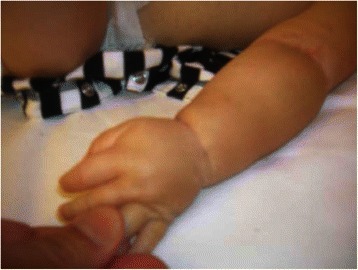
Fovea sign

After about 2 months of this diet, the child began to reject the food, in particular solid foods and to suffer from dysphonia and dysphagia due to the occurrence of laryngeal edema. Because of this the child was given only rice milk. After a few days the edema appeared on his feet, legs and upper extremities followed by a reduced urine output. He had no symptoms of gastroesophageal reflux, but he had forceful vomiting. When hospitalized, he was in a poor clinical condition with generalized edema (Fig. 1) and low urine output. He weighed 7.600 Kg from the age of six months up to 1 year. Blood and urine findings were normal except for the following results: total protein 3 g/dl; albumin 1.365 g/dl (45.5 %); total serum IgE 30 KUA/L; specific serum IgE: milk 0.64 KUA/L; albumen 1.74 KUA/L. Protein was not found in the urine. He required central access due to difficulty obtaining peripheral access due to severe edema. He was also found to be anemic with a haemoglobin 5.7 g/dl and he received 4 g of albumin three times in 48 h, a red blood cells transfusion, oral iron and folic acid. Vitamin K was also supplied because of a state of coagulopathy [activated partial thromboplastin: 31 s; prothrombin time 69 % (normal value: 80–100 %)]; fibrinogen 139 mg/dl; antitrombin III: 65 % (normal value: 80–100 %)]. The child was immediately fed with cow’s milk, which was well tolerated. Guidance from a nutrionist was essential and the edema gradually resolved.

After few days diuresis increased and weight initially decreased. Eczema improved and scratching was less evident thanks to topical treatment. Haematic examinations performed on day 10 showed a normalization of total protein, albumin and clotting tests. On follow up the skin prick test was negative to milk and egg allergy.

Because of a long lasting low blood calcium level (7.3 mg/L), he developed demineralization of his teeth, persisting at the follow up visits up at 2 years of age.

This kwashiorkor case highlights the potential danger of inappropriate elimination diets in infants with AD, and illustrates the need for careful nutritional guidance in the management of AD. The use of rice milk resulted in hypoalbuminemia and poor weight gain.

Eczema is a chronically relapsing inflammatory skin disease and one of the most common skin disorders affecting up to 17 % of children [1], and it can rarely be managed with dietary changes alone.

Previous studies described similar cases [2–5], showing that alternative to cow’s milk such as rice in spite of fortification are not a sufficient protein source.

The very first case was described by Carvalho NF et al. in 2001 [3]. In 2003 Novembre et al. described a similar case [4] and after more than 10 years the same mistake is still not so rarely made.

## Conclusions

This case reinforces the concept that hypoallergenic diets should be managed by allergists with experience in food allergies. Consultation and consensus should be achieved between specialists in pediatrics, allergy, nutrition and endocrinology, before adopting severely restrictive diets. Consequently, the choice of an elimination diet should be limited to children with moderate to severe eczema not controlled by topical steroids, under strict nutritional surveillance [6–8].

### Consent

Written informed consent was obtained from the patient for the publication of this Case Report and any accompanyng images. A copy of the written consent is available for review by the Editor-in-Chief of this Journal.

## Abbreviations

AD: Atopic dermatitis
